# Modeling the Kinetics, Curing Depth, and Efficacy of Radical-Mediated Photopolymerization: The Role of Oxygen Inhibition, Viscosity, and Dynamic Light Intensity

**DOI:** 10.3389/fchem.2019.00760

**Published:** 2019-11-13

**Authors:** Jui-Teng Lin, Hsia-Wei Liu, Kuo-Ti Chen, Da-Chuan Cheng

**Affiliations:** ^1^New Vision, Inc., Taipei City, Taiwan; ^2^Department of Life Science, Fu Jen Catholic University, New Taipei City, Taiwan; ^3^Graduate Institute of Applied Science and Engineering, Fu Jen Catholic University, New Taipei City, Taiwan; ^4^Department of Biomedical Imaging and Radiological Science, China Medical University, Taichung, Taiwan

**Keywords:** cross-link, curing, efficacy, kinetic modeling, oxygen, ultraviolet light

## Abstract

Kinetic equations for a modeling system with type-I radical-mediated and type-II oxygen-mediated pathways are derived and numerically solved for the photopolymerization efficacy and curing depth, under the quasi-steady state assumption, and bimolecular termination. We show that photopolymerization efficacy is an increasing function of photosensitizer (PS) concentration (*C*_0_) and the light dose at transient state, but it is a decreasing function of the light intensity, scaled by [*C*_0_/*I*_0_]^0.5^ at steady state. The curing (or cross-link) depth is an increasing function of *C*_0_ and light dose (time × intensity), but it is a decreasing function of the oxygen concentration, viscosity effect, and oxygen external supply rate. Higher intensity results in a faster depletion of PS and oxygen. For optically thick polymers (>100 um), light intensity is an increasing function of time due to PS depletion, which cannot be neglected. With oxygen inhibition effect, the efficacy temporal profile has an induction time defined by the oxygen depletion rate. Efficacy is also an increasing function of the effective rate constant, *K* = *k*′/$k_{\text{T}}^{0.5}$, defined by the radical producing rate (*k*′) and the bimolecular termination rate (*k*_T_). In conclusion, the curing depth has a non-linear dependence on the PS concentration, light intensity, and dose and a decreasing function of the oxygen inhibition effect. Efficacy is scaled by [*C*_0_/*I*_0_]^0.5^ at steady state. Analytic formulas for the efficacy and curing depth are derived, for the first time, and utilized to analyze the measured pillar height in microfabrication. Finally, various strategies for improved efficacy and curing depth are discussed.

## Introduction

Photoinitiated (photosensitized) polymerization and cross-linking provide advantageous means over thermal-initiated polymerization, including fast and controllable reaction rates, and spatial and temporal control over the formation of the material, without the need for high temperatures or harsh conditions (Fouassier, 1995; Odian, 2006). Tissue engineering using scaffold-based procedures for chemical modification of polymers has been reported to improve its mechanical properties by cross-linking or polymerization with ultraviolet (UV) or visible light to produce gels or high-molecular-weight polymers (Chen and Shi, 2014; Kotisch et al., 2017). The progress of light-responsive smart nanomaterials was recently review by Yang et al. (2018).

Industrial applications include developing materials for applications such as thin films, coatings, printing, graphic work, dentistry, contact lenses, and electronics. It is a noncontact, low-energy, and rapid process with capabilities of spatially specifying the reaction via photomasks (photolithography) (Cabral et al., 2004; O'Brien and Bowman, 2006; Cramer et al., 2008; Dendukuri et al., 2008; Alvankarian and Majlis, 2015; Wohlers and Caffrey, 2016; Chen et al., 2017; Wu et al., 2018). The polymerization rate is inhibited by air due to oxygen inhibition, which scavenges the radical species needed for cross-linking initialization. Diffusion of oxygen from a high-concentration zone into a prepolymer resin during UV curing requires an additional amount of photoinitiator and UV energy to consume the dissolved and diffused oxygen (O'Brien and Bowman, 2006; Cramer et al., 2008; Dendukuri et al., 2008; Alvankarian and Majlis, 2015).

Cabral et al. (2004) investigated the concept of frontal photopolymerization, in which the position of the frontal border can be controlled by adjusting UV power and the available atmospheric air. The utilization of microfabrication to reduce the deposition steps and to obtain a monolithic product was reported by Alvankarian and Majlis (2015). Chen et al. (2017) proposed a kinetic model for pillar growth that includes free-radical generation and oxygen inhibition in thick films of photoinitiated media and have demonstrated control over the pillar spacing and pillar height with the irradiation intensity, film thickness, and the size and spacing of the optical beams. In microfabrication system, the formation of radical decreases over depth due to the reduction in light intensity and photosensitizer (PS) concentration and increase in oxygen inhibition. Oxygen diffuses into the film and consumes radicals. The balance of radical production and oxygen inhibition gives rise to the inhibition zone, where the polymerization is completely suppressed (Wu et al., 2018).

The kinetics of polymerization has been extensively studied (Cabral et al., 2004; O'Brien and Bowman, 2006; Cramer et al., 2008; Dendukuri et al., 2008; Alvankarian and Majlis, 2015; Semchishen et al., 2015; Lin and Wang, 2016; Chen et al., 2017; Lin and Cheng, 2017; Lin, 2018, 2019; Wu et al., 2018; Lin et al., 2019). However, most of the previous models (Cabral et al., 2004; O'Brien and Bowman, 2006; Cramer et al., 2008; Dendukuri et al., 2008; Alvankarian and Majlis, 2015; Chen et al., 2017; Wu et al., 2018) are based on oversimplified assumptions of constant PS concentration (without depletion), and the light intensity in the polymer following a conventional Beer–Lambert law (BLL), with neglected PS depletion, which is only valid for optically thin polymers (Lin and Cheng, 2017).

This study will present a more general kinetic model with coupled equations for the PS and oxygen concentration and monomer, which will be numerically solved for various conditions. Analytic formulas for the efficacy and curing depth will be derived and utilized to analyze the measured pillar height in microfabrication reported by Chen et al. (2017). Various scaling laws for the efficacy and curing depth, in both transient and steady state, will be derived, for the first time. The importance of PS depletion and dynamic light intensity in an optically thick polymer, based on a revised BLL, will be shown numerically (Lin and Cheng, 2017). Finally, various strategy for improved efficacy and curing depth, by reducing oxygen inhibition effect, will be discussed.

## Methods

### Kinetic Equations

Photopolymerization in general includes radical-mediated, cationic, and anionic catalyzed, and atom transfer radical polymerization. In a radical-mediated photopolymerization, the monomer is converted to polymer after the light irradiation of the photosensitizer (PS) or photoinitiator. The UV (or visible) light-produced triplet state (T^*^) is coupled to the substrate monomer [A] and the oxygen to produce radicals. Each radical becomes the center of origin of a polymer chain. The chain growth of a polymer radical with m-links stops as a result of chain termination reactions. Kinetic equations of an m-component radical photopolymerization process (with a triplet excited state as the catalyst) may be described as follows: (Semchishen et al., 2015; Lin, 2019)

(1) $$\frac{\partial T(z,t)}{\partial t}\;=bIC-(k_{5}+k_{3}[{\text{O}}_{2}]+k_{7}[\text{A}])\text{T}$$

(2) $$\frac{\partial [{\text{O}}_{2}]}{\partial t}=P-k_{3}T[{\text{O}}_{2}]+\;-\sum_{m=1}^{\infty }\;k_{m}R_{m}[{\text{O}}_{2}]$$

(3) $$\begin{array}{rcl} \frac{\partial R_{1}}{\partial t} & = & k_{8}\left[\text{A}\right]\text{T}-{\text{k}}^{\prime }{\text{R}}_{1}\left[\text{A}\right]-{\text{R}}_{1}\sum_{\text{m}=1}^{\infty }{\text{ k}}_{\text{m}}{\text{R}}_{\text{m}} \end{array}$$

(4) $$\begin{array}{rcl} \frac{\partial R_{n+1}}{\partial t} & = & k^{\prime }\left(R_{n}-R_{n+1}\right)\left[\text{A}\right]-R_{n+1}\sum_{m=1}^{\infty }{\;k}_{m}R_{m} \end{array}$$

(5) $$\begin{array}{rcl} \frac{\partial \left[\text{A}\right]}{\partial t}\; & = & -k_{7}\left[\text{A}\right]T-\sum_{m=1}^{\infty }{\;k}_{m}R_{m}\left[\text{A}\right] \end{array}$$

**Figure 1:**
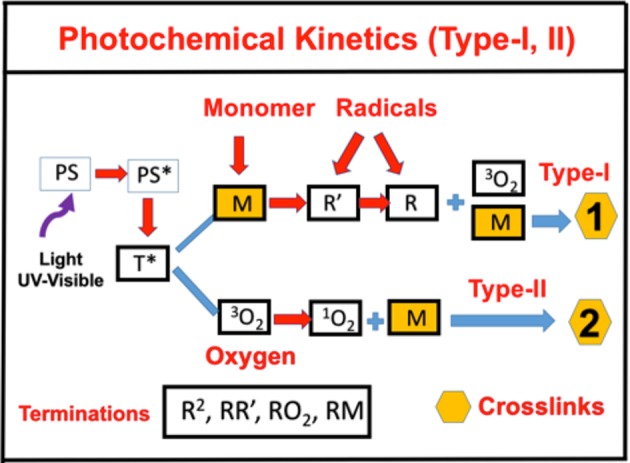
Schematics of photochemical pathways. Pathway of one-monomer system, for radical-mediated pathway-1 and oxygen-mediated pathway-2; where *P* is the ground-state photosensitizer, having an excited and triplet state PS* and T*, which interacts with the substrate A to form radicals *R*′ and *R*.

As shown in Figure 1, for a one-component monomer A, with three-radical system consisting of two PS radicals (*R*′ and *R*) and one singlet oxygen, the pathways are described as follows. The ground-state PS molecules are excited by the UV light to its singlet excited state (*S*_1_), which could be relaxed to its ground state or to a triplet excited state (T^*^). In type-I process, T^*^ could interact directly with the substrate [A] and produces the first-radical (*R*′), which could produce (by chain reaction) a second radical (*R*) which could interact with the ground state oxygen or the first radical. For type-II process, (*T*_3_) interacts with [O_2_] to form oxygen singlet [^1^O_2_]. These reactive radicals, *R, R*′, and [^2^O_1_], could be relaxed to its ground states or interacts with the substrate [A]. This article will focus on the one-monomer system, and the two-monomer and two-initiator systems will be shown elsewhere (Chen et al., 2019b; Lin, 2019).

**Figure 2:**
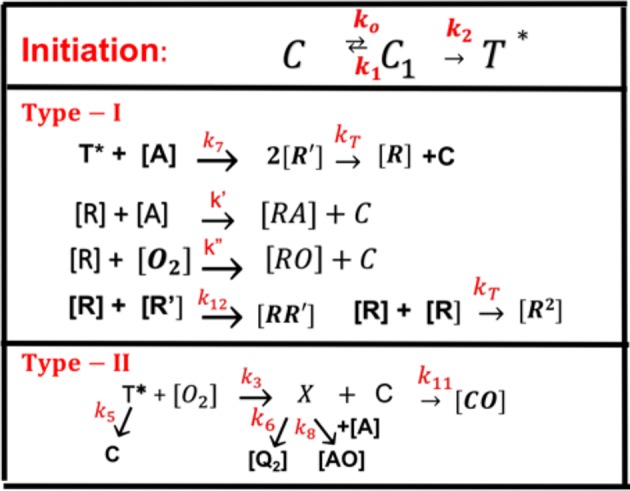
The ground-state photosensitizer (PS) (*C*) is excited by a UV light to its singlet excited state (*C*_1_), which could be relaxed to its ground state or to a triplet excited state (T*). In type-I process, T* could interact directly with the monomer [A] to generate free radical (*R*′) by recombination. The radical *R* could interact with [A] for cross-linking, or oxygen [O_2_], or terminated by coupling with *R*′, or bimolecular recombination (2*R*^2^). For type-II process, T* interacts with [O_2_] to form oxygen singlet [^1^O_2_] (*X*), which could relax to its oxygen [O_2_], or interacts with [A] for cross-linking, or coupling with *C*. All rate constants are shown in reds next to arrows.

For a one-monomer system, using the short-hand notations, the concentration of various components: *C* (*z, t*) and *T* for the PS ground and triplet state, respectively; [O_2_] and *X* for the oxygen ground and singlet state; *R* and *R*′ for the first and intermediate free radical; and [A] for the available extracellular matrix substrate; and the kinetic equations for a three-radical system becomes

(6) $$\frac{\partial C(z,t)}{\partial t}\;=-\;bI\left(z,t\right)C-\;k_{11}XC+(k_{5}+k_{3}[{\text{O}}_{2}])T+R_{E}$$

(7) $$\frac{\partial T}{\partial t}\;=bI\left(z,t\right)C-\;(k_{5}+k_{3}[{\text{O}}_{2}]+k_{7}[\text{A}])T$$

(8) $$\begin{array}{rcl} \frac{\partial R^{\prime }}{\partial t}\; & = & 2k_{7}T\left[\text{A}\right]-\;{\text{k}}_{12}\text{R}{\text{R}}^{\prime }-{2\text{k}}_{\text{T}}{{\text{R}}^{\prime }}^{2} \end{array}$$

(9) $$\begin{array}{l} \frac{\partial R}{\partial t}\;=2k_{T}{R^{\prime }}^{2}-kR\left[\text{A}\right]-\;k_{12}RR-\;2k_{T}R^{2}\\ \;-k^{\prime \prime }R[{\text{O}}_{2}] \end{array}$$

(10) $$\frac{\partial X}{\partial t}\;=\;k_{3}[{\text{O}}_{2}]T-(k_{6}+k_{11}C+k_{8}[\text{A}])X$$

(11) $$\frac{\partial \;[{\text{O}}_{2}]\;}{\partial t}=P\;-\;k_{3}[{\text{O}}_{2}]T+k_{6}X-k^{\prime \prime }R[{\text{O}}_{2}]$$

(12) $$\frac{\partial [\text{A}]}{\partial t}\;=-(k_{7}T+k_{8}X+\;k^{\prime }R)[\text{A}]$$

where *b* = 83.6*a*′*qw, w* being the UV light wavelength (in cm) and light intensity *I*(*z,t*) in mW/cm^2^; q is the quantum yield of the PS triplet state; Equation (11) also includes an oxygen source term given by Semchishen et al. (2015), *P* = (1 – [O_2_]/O_0_)*P*′, with a maximum rate constant *P*′, with a maximum rate constant *P*_0_ and initial oxygen concentration *Y*_0_. This term may be also given by the oxygen diffusion *P* = $D_{0}\nabla^{2}\left[O\right].$ In Equation (1), the regeneration term is given by *R*_E_ = *k*″*R*[O_2_] + 2*k*_*T*_(*R*^2^ + *R*^′2^),

All the reaction rate constants are defined by the associated coupling terms, as shown by Figure 2. For examples, in Equation (9), *k*_7_ is for the coupling rate of [A] and *T*, which has a ground-state relaxation rate *k*_5_; in Equation (11), *k*′ is for [A] and *R*, which is coupled with *R*′ by *k*_12_ and a bimolecular termination rate of *k*_*T*_; in Equation (12), *k*′ is for the reaction of [A] and *R*, which is coupled with oxygen by *k*″ and a bimolecular termination rate *k*_*T*_. Figure 2 also shows the rate constants for type-II process: *k*_3_ for singlet-oxygen (*X*) production; *k*_8_ for cross-link of *X* with [A] and having a relaxation rate of *k*_6_; and a reduction rate *k*_11_, by coupling to C.

The dynamic light intensity is given by (Lin and Wang, 2016; Lin and Cheng, 2017)

(13) $$\begin{array}{rcl} \frac{\partial I\left(z,t\right)}{\partial z}\; & = & -A^{\prime }\left(z,t\right)I\left(z,t\right) \end{array}$$

(14) $$\begin{array}{rcl} A^{\prime }\left(z,t\right) & = & 2.3\left[\left(a^{\prime }-b^{\prime }\right)C\left(z,t\right)+b^{\prime }C_{0}+Q\right] \end{array}$$

where *a*′ and *b*′ are the extinction coefficients of PS and the photolysis product, respectively; *Q* is the absorption coefficient of the monomer at the UV wavelength. Most previous modeling (Cramer et al., 2008; O'Brien and Bowman, 2006; Dendukuri et al., 2008; Alvankarian and Majlis, 2015; Chen et al., 2017; Wu et al., 2018) assumed a constant *C* (*z, t*) in Equation (14). Our analytic formulas in this article will use a time average of *A* (*z, t*) to count for the dynamic of light intensity due to PS depletion.

The kinetic Equations (6)–(12) may be numerically calculated to find the conversion efficacy, which, however, is too complex for us to analyze the roles of each of the parameters. For comprehensive modeling, we will use the so-called quasi-steady-state assumption (Lin, 2018; Lin et al., 2019). The life time of the singlet and triplet states of photosensitizer, the radicals (*R* and *R*′), and the singlet oxygen (*X*) are very short (nanosecond to microsecond time scale) since they either decay or react with cellular matrix immediately after they are created. Thus, one may set d*T*/d*t* = d*R*/d*t* = d*R*′/d*t* = d*X*/d*t* = 0, which give the quasi-steady-state solutions: *T* = *bIgC, X* = *bIg*′ [O_2_]/*k*_8_, *g* = *k*_8_/(*k*_3_[O_2_] + *k*_8_[A] + *k*_5_); *g*′ = *k*_3_/(*k*_6_ + *k*_11_*C* + *k*_7_[A]);_._ *k* = (*k*_11_/*k*_8_). The kinetic Equations (8)–(12) become,

(15) $$\frac{\partial C(z,t)}{\partial t}=-bI(g[\text{A}]+kgg^{\prime }C[{\text{O}}_{2}])C+R_{E}$$

(16) $$\frac{\partial [{\text{O}}_{2}]}{\partial t}=-(bICg+k^{\prime \prime }R)[{\text{O}}_{2}]+P$$

(17) $$\frac{\;\partial [\text{A}]}{\partial t}=-{\left[bIgC\left(1+g^{\prime }\left[{\text{O}}_{2}\right]\right)+k^{\prime }\;R\right]}_{\;}\left[\text{A}\right]$$

where the PS regeneration term *R*_E_ = *k*_9_*R*[O_2_] + 2*k*_*T*_*R*^2^. The radicals, *R* and *R*′, are given by the solution of the following steady-state of Equations (8) and (9):

(18) $$\begin{array}{r} 2bIgC\left[\text{A}\right]-\;k_{12}RR^{\prime }-{2k}_{T}{R^{\prime }}^{2}=0 \end{array}$$

(19) $$2k_{T}{R^{\prime }}^{2}-\;k^{\prime }R\left[\text{A}\right]-k_{12}RR^{\prime }-2k_{T}\;R^{2}-k^{\prime \prime }R[{\text{O}}_{2}]=0\;$$

Numerical solutions are required for *R* and *R*′. However, analytic formulas are available for the case that coupling of *R*′ and *R* is weaker than the bimolecular recombination, i.e., 2*k*_*T*_*R*^′2^ >> *k*_12_*RR*′ in Equation (18), we obtain *R*′ = (*bIgC*[A]/*k*_*T*_)^0.5^. Substituting this steady-state *R*′ into Equation (19), we obtain

(20) $$R\left(z,t\right)=\left(\frac{1}{4\;k_{T}}\right){\left[-G+\sqrt{G^{2}+16k_{T}B(z,t)}\;\right]}_{\;}$$

where *B* = *bICg*[A], and *G* = *k*″ [O_2_] + 2*k*_12_*R*′ + *k*′[A]. For *G*^2^ << 8*k*_*T*_*B*, Equation (20) is further reduced to *R* = (*B*/*k*_*T*_)^0.5^ – *k*″ [O_2_](1 – *B*′)/(4*k*_*T*_), with *B*′ = 0.5*k*″[O_2_]/(8*Bk*_*T*_)^0.5^, which shows that *R*, and efficacy, are decreasing functions of *k*″ [O_2_], referred as the oxygen inhibition effect.

Equations (15)–(17) may be numerically solved. However, it requires all the rate constants (*k*_*j*_) to be given for specific materials. Without knowing these parameters, we will further simplify Equations (15)–(17) as follows. In most situations, the monomer concentration ([A]) is much larger than the oxygen, which is also depleted faster than PS (or *C*), or *k*_8_[A] >> (*k*_3_[O_2_] + *k*_5_), such that *g* = 1/[A], *k*″[O_2_]*C*/*A*^2^ << 1, and *bIC*/*A* << *k*′*R*. These conditions were also reported by Dendukuri et al. (2008), Alvankarian and Majlis (2015), and Chen et al. (2017) in their modeling. Therefore, the depletion of *C* of Equation (15) is mainly due to type-I mechanism, whereas oxygen depletion is mainly due to the coupling term of *k*″*R*[O_2_] in Equation (16). Equations (15)–(17) reduce to the following

(21) $$\begin{array}{rcl} \frac{\partial C}{\partial t} & = & -\;bI\left(z,t\right)C\left(z,t\right) \end{array}$$

(22) $$\frac{\partial [\text{O}]}{\partial t}=\;-k^{\prime \prime }R\left(z,t\right)[{\text{O}}_{2}]+\;P\;$$

(23) $$\begin{array}{rcl} \frac{\partial \left[A\right]}{\partial t} & = & {-k^{\prime }R\left(z,t\right)\left[\text{A}\right]}_{\;} \end{array}$$

The above simplified kinetic equations reduce to that of Dendukuri et al. (2008) for the special situations (or assumptions): the PS concentration, *C*(*z,t*), is a constant, or Equation (21) d*C*/d*t* = 0 (or *bIt* << 1, for small dose), and light intensity is also a constant, or Equation (13), d*I*/d*z* = 0, which, however, is valid only for a short exposure time, or an optically thin polymer (with *Az* << 0.1). Wu et al. (2018) included the light intensity reduction in their thick polymer system. However, they have ignored the PS depletion, or d*C*/d*t* = 0. Furthermore, the rate constants, *k*′ and *k*″, in general, are reduction function of the monomer conversion efficacy due to the viscosity effects, which were also ignored by Dendukuri et al. (2008) and Alvankarian and Majlis (2015). They also used a simplified format of Equation (16) for the oxygen inhibition effect. Our Equations (15)–(17), to be numerically solved later, are more accurate than previous model (Dendukuri et al., 2008; Alvankarian and Majlis, 2015; Semchishen et al., 2015; Chen et al., 2017; Wu et al., 2018), which used our simplified Equations (22) and (23), with *I*(*z,t*) and *C*(*z,t*) are constants.

Accurate solutions of Equations (21)–(23) require numerical simulations (to be shown later). For analytic formulas, we will use approximated analytic formulas for the light intensity and the PS concentration, such that we do not need to solve for Equation (13), and the expressive closed forms of *I*(*z,t*) and *C*(*z,t*) allow us to solve for the first- and second-order solutions of [O_2_], [A], and the conversion efficacy.

### Analytic Formulas for Efficacy

The monomer conversion efficacy for a bimolecular termination process is given by *C*_EFF_ = 1 – [A]/[A]_0_ = 1 – exp(–S), with [A_0_] being the initial monomer concentration, and the S-function is given by the time integral of *k*′R. Without the oxygen diffusion (with *P*′ = 0), the first-order solution of Equation (22), with *R* = (0.5*B*/*k*_*T*_)^0.5^ is given by, [O]^(1)^ = *Y*_0_ exp(–S), *Y*_0_ is the initial oxygen, which gives the second-order solution of Equation (6), *R*(2) = *B*/$k_{\text{T}}^{0.5}$ – *k*″ [O_2_](1 – *B*′)/(4*k*_*T*_), with *B* = *bIC, B*′ = 0.5*k*″ [O_2_]/(*Bk*_*T*_)^0.5^, for *B* << 1. *R*′ (2) may be used to find the second-order solution of oxygen concentration (for *P*′ = 0) given by

(24) $$\left[{\text{O}}_{2}]\;=Y_{0}\exp(-S^{\prime }\right)$$

(25) $$\begin{array}{rcl} S^{\prime } & = & k^{\prime }\int_{0}^{t}R^{\prime }\left(2\right)dt \end{array}$$

To include the oxygen diffusion effect, for small time when oxygen is present, the approximate solution of Equation (22) is given by (Dendukuri et al., 2008)

(26) $$\left[{\text{O}}_{2}]\;=Y_{0}-4k^{\prime }Bt/(\text{π}k\right)$$

The induction time (*T*_ID_) is defined by when [O_2_] = 0. We obtain

(27) $$\begin{array}{r} T_{ID}=\pi kY_{0}/\left(4k^{\prime }B\right) \end{array}$$

During the induction time (with *t* < *T*_ID_), the solution of Equation (23) is given by

(28) $$\begin{array}{rcl} \left[\text{A}\right] & = & A_{0}\exp\left(-S^{\prime }\right) \end{array}$$

(29) $$\begin{array}{rcl} S^{\prime } & = & k^{\prime }\int_{T_{ID}}^{t}\left(B/G^{\prime }\right)dt^{\prime } \end{array}$$

where *G*′ = *D*_0_*Y*_0_(1 – 4*k*′*Bt*/(π*k*).

For large time when oxygen is largely depleted, the radical concentration is given by *R* = (*B*/*k*_*T*_)^0.5^ – *K*_12_[O_2_], with *K*_12_ = *k*′(1 – *B*′)/(4*k*_*T*_), with [O_2_] given by Equation (24), which gives the solution of Equation (23),

(30) $$\begin{array}{rcl} \left[\text{A}\right] & = & A_{0}\exp\left(-S\right) \end{array}$$

(31) $$S=k^{\prime }\int_{0}^{t}(\sqrt{0.5B(z,t)/k_{T}\;}-K_{12}[{\text{O}}_{2}])dt^{\prime }$$

where [O_2_] may be analytically given by Equations (24) or (26). Equation (30) was also presented by Alvankarian and Majlis (2015). However, they assumed a constant PS concentration, or B is time independent. When the diffusion and consumption of oxygen inside the photo resin, or d[O_2_]/d*t* = 0, the closed form solution is available. For the case that oxygen diffusion is stopped, or d[O_2_]/d*z* = 0, the closed form solution of oxygen may be used to find the closed form solution of Equation (22) and the conversion efficacy. We note that, during the induction time (with *t* < *T*_*ID*_), *S*′ of Equation (29) is much smaller than *S* of Equation (31); therefore, the conversion efficacy is a fast-rising function of time after *t* > *T*_*ID*_. We will show the numerical results later.

### The Dynamic Light Intensity

Solving Equations (13) and (14) for the light intensity, *I*(*z,t*) and PS concentration *C*(*z,t*) concentration, we may numerically find *S*′ and then the conversion efficacy. We may further derive the analytic form of conversion efficacy which requires a closed form of *I*(*z,t*) and *C*(*z,t*) as follows. Using our previously developed approximated analytic formulas (Lin and Wang, 2016; Lin and Cheng, 2017)

(32) $$\begin{array}{rcl} I\left(z,t\right) & = & I_{0}\exp\left[-A^{\prime }z\right] \end{array}$$

(33) $$\begin{array}{rcl} C^{\prime }\left(z,t\right) & = & C_{0}\exp\left[-B^{\prime }t\right] \end{array}$$

(34) $$\begin{array}{rcl} A^{\prime }\left(z,t\right) & = & 2.3\left(a^{\prime }C_{0}+Q\right)-A_{1}t \end{array}$$

where *B*′ = *bI*_0_exp(–*A*″*z*), *A*_1_ = 2.3(*a*′ – b′)*C*_0_*I*_0_*bz*, with *A*″ being the averaged absorption given by *A*″ = 0.5 × 2.3(*a*′ + *b*′) + 2.3*Q*. We note that the –*A*_1_*t* term represents the decrease in *A*′ or increase in light intensity due to PS depletion. Using Equations (32)–(34), we obtain analytic solution of Equation (30), when *K*_12_[O_2_] is ignored in Equation (31) for a type-I predominated mechanism, as follows.

(35) $$\begin{array}{rcl} S & = & KG\left(z,t\right)\sqrt{0.5bXI_{0}C_{0}} \end{array}$$

(36) $$\begin{array}{rcl} G\left(z,t\right) & = & \left[1-\exp\left[-B^{\prime \prime }t^{\prime \prime }\right)\right]/B^{\prime \prime } \end{array}$$

where *K* = *k*′/*k*_*T*_^0.5^, *t*″ = *t – T*_*ID*_, *B*″ = 0.5(*B*′ – 0.5*A*_1_*t*), *X* = exp[–*A*_2_*z*], with *A*_2_ = 2.3(*a*′*C*_0_ + *Q*). We note that Equation (34) defines the dynamic feature of the light intensity which is an increasing function of time due to the depletion of the PS concentration. It also provides the non-linear dynamic dependence of *A*′(*z,t*), given by *A*_1_*t*, which is important for optical-thick polymer and for high light dose. Greater detail analysis will be given later. The above analytic formulas provide useful information to analyze and predict critical roles of each of the influencing factors without numerically solving the coupled equations. Equation (35) will also be used to analyze the numerical results and the experimental data later.

### The Effects of Viscosity

To include the viscosity effects, the free-volume is reduced when cross-link efficacy increases. As proposed by Wu et al. (2018), the rate parameters *k*′ and *k*_*T*_, are decreasing function of the efficacy (*C*_EFF_) given by (in CGS units, cm^3^/mM/s): *k*′ = 1,865/(1 + 2*E*′ × 10^−9^), *k*_*T*_ = 10^7^/(2.3 + *E*′) + *k*′(1 – *C*_EFF_); with *E*′ = exp(*vC*_EFF_), where *v* is a constant ranging 25–40 defining the viscosity strength. High viscosity effect leads to a lower conversion efficacy. To include the viscosity effect, the effective rate constant, *K* = *k*′/*k*_*T*_^0.5^, of Equation (14) may be revised to a smaller value as: 1 – m [1 – exp(–*S*)], with *S* is the first-order solution (with no revision), where *m* = 0.1–0.3 is a fit parameter to measured data. The revised *K* leads to a smaller efficacy due to the less free volume of cross-link resulted by the increase in viscosity when efficacy increases. Numerical results will be shown later.

### Curing Depth and Inhibition Zone

A curing depth (*Z*_C_) is defined by when the conversion efficacy is higher than a critical value, *C*_EFF_ > *C*_*T*_, or when *S* > *S*_*T*_, with *S*_*T*_ = ln [1/(1 – *C*_*T*_)]. Using Equation (35), and let *S* = *S*_*T*_ = 2 (or *C*_*T*_ = 0.86), *Z*_*C*_ is related to the cross-link time (*T*_*C*_) by, for the case that *K*_12_[O_2_] is ignored in Equation (31),

(37) $$\begin{array}{r} T_{C}=\left(\frac{1}{B^{\prime \prime }}\right)\ln[2B^{\prime \prime }/(K^{\prime }\sqrt{0.5bX^{\prime }I_{0}C_{0}}\;)-1\;] \end{array}$$

where *K*′ = *k*′/*k*_*T*_^0.5^, *X*′ = exp(–*A*″*Z*_*C*_) defines the curing depth (*Z*_*C*_). We plot the curve of *T*_*C*_ vs. *Z*_*C*_, then rotate the axis to show curve of *Z*_*C*_ vs. *T*_*C*_. The above curing depth (*Z*_*C*_) is proportional to the pillar height measured by Chen et al. (2017) where their measured data of **Figure 4** will be analyzed by Equation (37) later. For a given photo resin thickness of *H*, Chen et al. (2017) also defined an inhibition zone (*Z*_*D*_) given by *Z*_*D*_ = *H* – *Z*_*C*_. When the oxygen inhibition effect, or *k*[O_2_] is included in Equation (31), the analytic formula of Equation (37) is not available and *Z*_*C*_ needs a numerical solution to be shown later.

## Results and Discussion

In the following numerical solutions of Equations (21)–(23), we will also use the radical *R* given by Equation (20) and light intensity given by Equation (32). The roles of key parameters of *b, k*″, *I*_0_, and the initial PS and oxygen concentrations, polymer thickness, and the viscosity effect will be analyzed.

### Dynamic Concentration and Light Intensity

**Figure 3:**
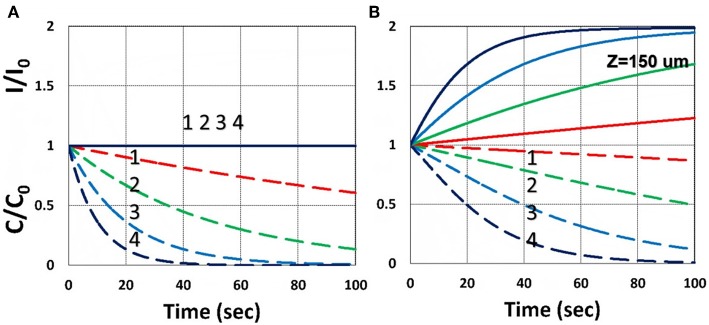
Dynamic profiles of the PS concentration (blue curves) and light intensity increase ratio (red curves) at various light intensity *I*_0_ = (0.5, 2, 5, 10) mW/cm^2^, at *z* = 0 **(A)** and 150 μm **(B)**.

Figure 3 shows the dynamic profiles of the PS concentration and light intensity obtained by the numerical solution of Equations (21)–(23), using light intensity given by Equation (32). Depending on the coupling parameter *b* and *I*(*z,t*), as shown by Equation (32), the depletion of *C*(*z,t*) causes the increasing profile of *I*(*z,t*), defined by a ratio *R*_*Z*_′ = *I*(*z,t*)/*I*(*z,t* = 0), which is also a decreasing function of the depth (*z*) per BLL. Figure 3 shows the ratio *R*_*Z*_, at *z* = 150 μm, for *b* = 0.01–0.02, *a*′ = 458 (1/mM), *b*′ = *Q* = 0. Figure 2 shows that higher intensity results a faster PS depletion and hence larger light intensity increase. For thick polymers (>100 μm). Therefore, the assumption of time-independent light intensity and PS concentration is valid only for optically thin (with *A*′*z* < 0.2) polymers and under a small dose, i.e., under the condition that the time-dependent term *A*_1_*t* of Equation (34) is neglected. For optically thick polymers under a larger dose, with *bI*_0_*tz* > 0.2, the thin-polymer assumption (O'Brien and Bowman, 2006; Cramer et al., 2008; Dendukuri et al., 2008; Alvankarian and Majlis, 2015; Chen et al., 2017; Wu et al., 2018) will cause an error of 10–30%, depending on the value of *A*′ in Equation (34). Figure 3B shows the dynamic feature of *I*(*z,t*)/*I*_0_ due to PS depletion for *z* > 0.

### Efficacy Temporal Profiles

We will now show the numerical results of the efficacy by solving Equations (21)–(23) using light intensity given by Equation (32). We will also show the dynamic profiles of oxygen. Roles of key parameters of *b, k*″, and *I*_0_ and the initial PS and oxygen concentrations, polymer thickness, and the viscosity effect will be analyzed.

**Figure 4:**
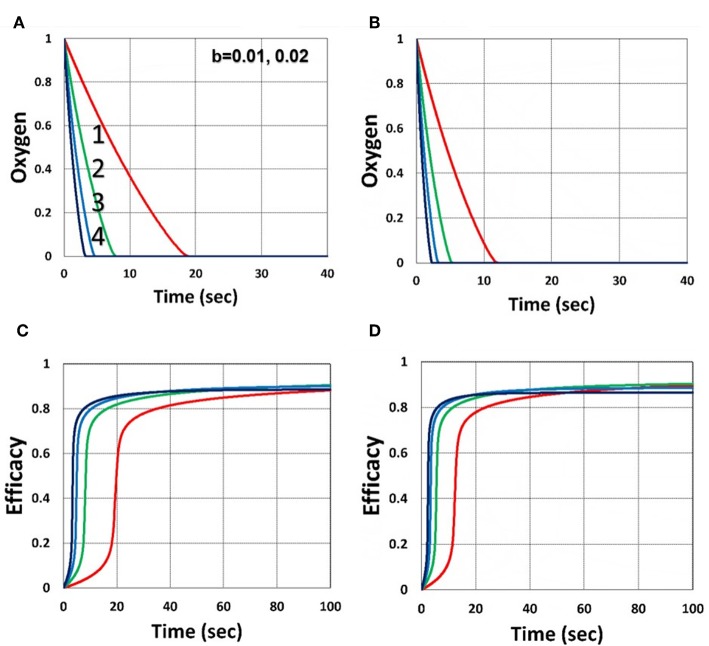
Dynamic profiles. Efficacy and oxygen profiles for various light intensity *I*_0_ = (0.5, 2, 5, 10) mW/cm^2^, for curves (1, 2, 3, 4), for *b* = 0.02, shown by **(A,C)**; and *b* = 0.04, shown by **(B,D)**; for *C*_0_ = 0.01 mM, and without viscosity effect (or *v* = 0), and without external oxygen supply (with *P*′ = 0).

**Figure 5:**
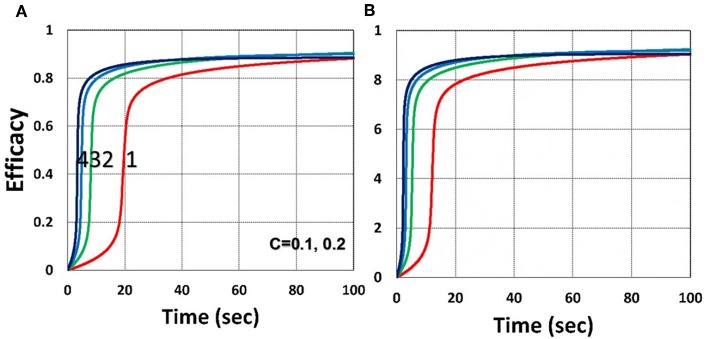
Dynamic profiles. Same as Figure 4, but for **(A)** *C*_0_ = 0.1% and **(B)** *C*_0_ = 0.2%; for *b* = 0.02.

As shown by Figure 4, higher light intensity (*I*_0_) and coupling factor (*b*) cause faster oxygen depletion (and shorter induction time) leading to higher conversion efficacy, as also predicted by Equations (24)–(35). Figure 5 shows that higher PS initial concentration (*C*_0_) leads to higher efficacy, as predicted by our analytic Equation (35).

**Figure 6:**
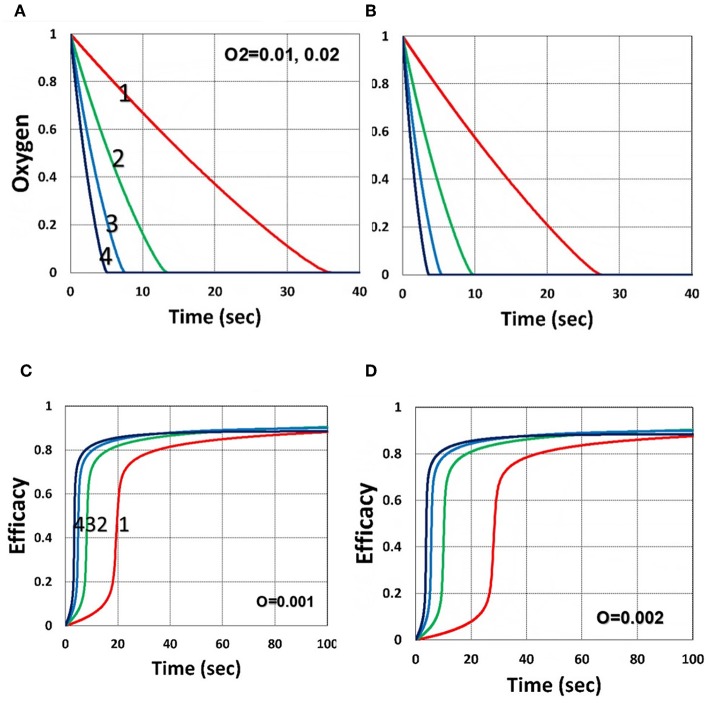
Dynamic profiles. Same as Figure 4, but for oxygen initial value of 0.001 mM shown by **(A,C)**; and 0.002 mM shown by **(B,D)**; for *b* = 0.02 and *C*_0_ = 0.01 mM.

Figure 6 shows that larger initial oxygen concentration causes higher inhibition effects and leads to lower efficacy, as predicted by Equation (31). It also shows longer induction time for lower light intensity and/or higher oxygen initial concentration. The induction time is defined by when oxygen is depleted.

**Figure 7:**
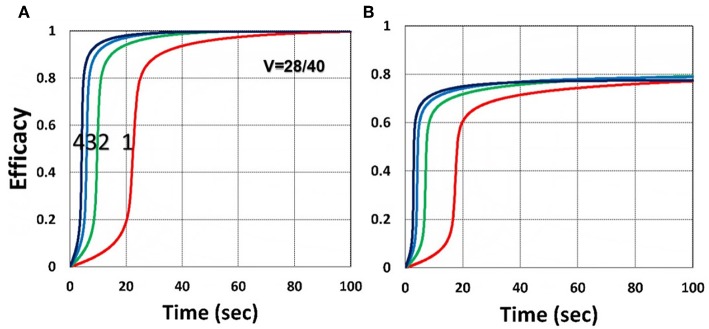
Dynamic profiles. Same as Figure 4, but for viscosity factor *v* = 28 **(A)**; and *v* = 40 **(B)**; for *b* = 0.02 and *C*_0_ = 0.01 mM.

**Figure 8:**
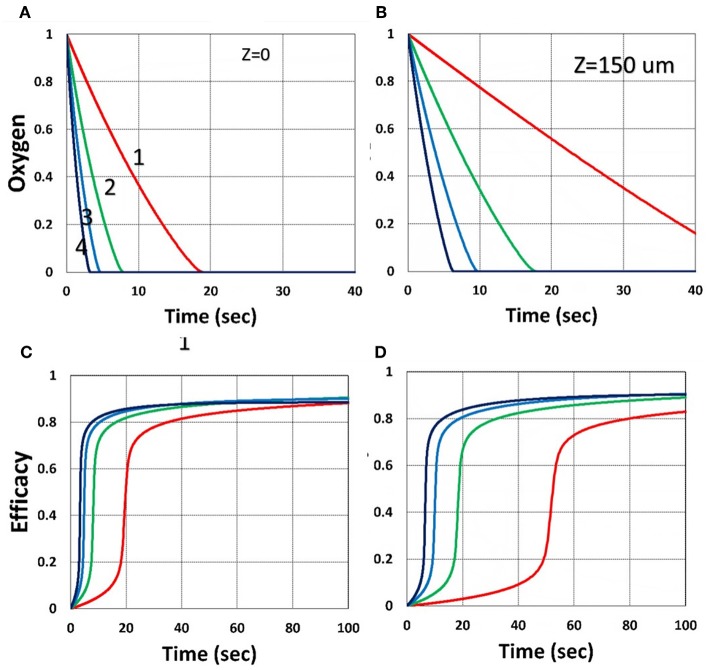
Dynamic profiles. Same as Figure 3, but for *z* = 0 (on surface), shown by **(A,C)**; and *z* = 150 μm, shown by **(B,D)**; for *b* = 0.02 and *C*_0_ = 0.01 mM.

Figure 7 shows that larger viscosity factor (*v*) causes smaller free volume and thus smaller radical coupling rate (*k*′) and leads to a lower efficacy, as predicted by the effective rate constant, *K* = *k*′/*k*_*T*_^0.5^, in Equation (35). Figure 8 shows that, inside the polymer (with *z* = 150 μm), both light intensity and PS concentration are reduced; thus, conversion is less, although oxygen is depleted slower inside the polymer, as predicted by the *z*-dependent *X* = exp[–*A*_2_*z*] in Equation (31).

**Figure 9:**
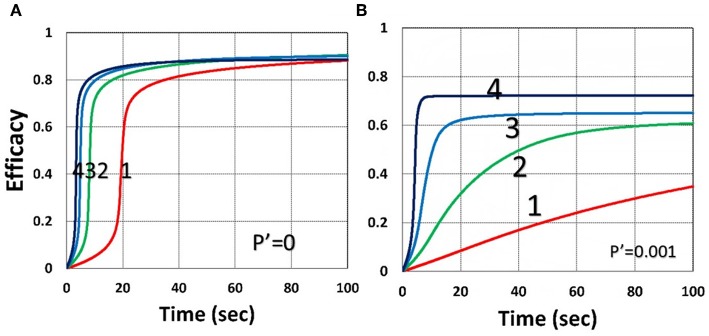
Dynamic profiles. Same as Figure 4, but for without (*P*′ = 0) and with (*P*′ = 0.001) external oxygen supply, shown by **(A,B)**, respectively; for *b* = 0.02 and *C*_0_ = 0.01 mM.

**Figure 10:**
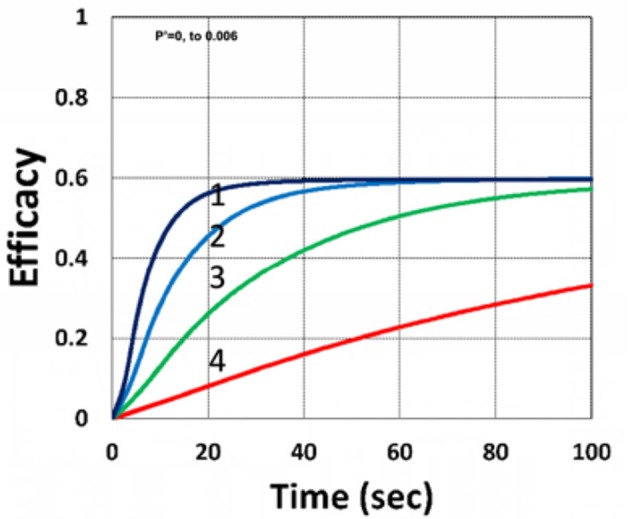
Dynamic profiles. Same as Figure 9, but for *P*′ = (0, 0.0001, 0.001, 0.006), for curves (1, 2, 3, 4); for *b* = 0.02, *I*_0_ = 5 mW/cm^2^ and *C*_0_ = 0.01 mM.

Figure 9 shows that external oxygen supply cause increased radical inhibition and thus smaller conversion, as predicted by Equation (34). Figure 10 shows the efficacy profiles for various external oxygen supply (*P*′ > 0) that larger *P*′ causes higher oxygen inhibition effect and leads to smaller efficacy, as predicted by Equation (31).

### Efficacy Spatial Profiles

**Figure 11:**
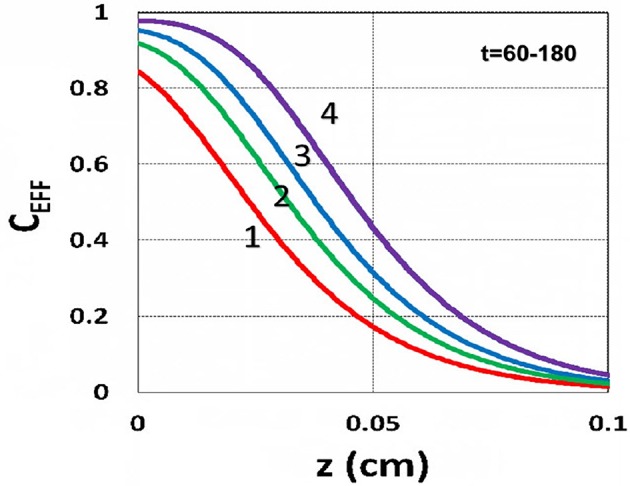
Efficacy spatial profiles. Efficacy vs. depth (*z*) for *t* = (60, 90, 120, 180) s, for curves (1, 2, 3, 4); for a fixed light intensity *I*_0_ = 15 mW/cm^2^, *C*_0_ = 0.1 mM, [O_0_] = 0.001 mM, *b* = 0.002, *A*′ = 1,000 (1/cm), in the absence of oxygen, based on Equation (35).

**Figure 12:**
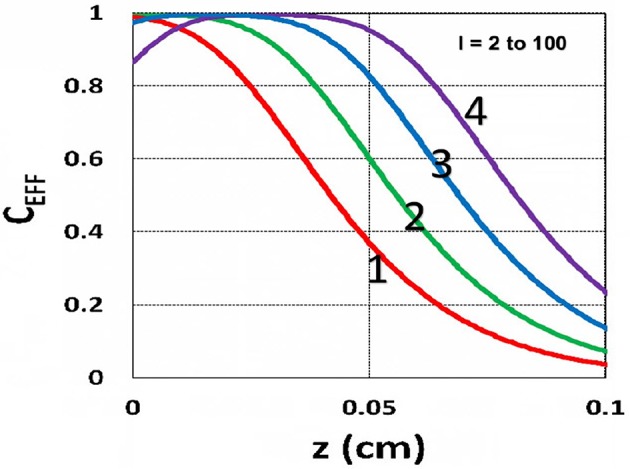
Efficacy spatial profiles. Same as Figure 11, but for *I*_0_ = (2, 8, 30, 100) mW/cm^2^, for curves (1, 2, 3, 4); for *t* = 400 s.

**Figure 13:**
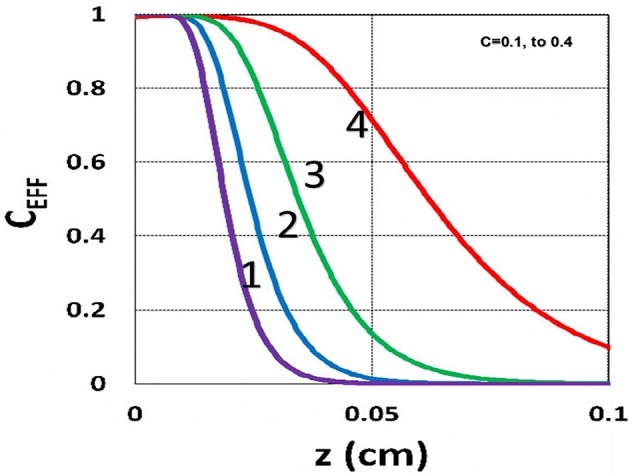
Efficacy spatial profiles. Same as Figure 11, but for *C*_0_ = (0.1, 0.2, 0.3, 0.4) mM, for curves (1, 2, 3, 4); for *I*_0_ = 15 mW/cm^2^, at *t* = 400 s.

The efficacy spatial profiles will be shown based on the analytic formula of Equation (35), with ignored oxygen effects, in Equation (31). Figure 11 shows that efficacy is a decreasing function of *z*, but it is an increasing function of light exposure time, as predicted by Equation (35). Figure 12 shows that efficacy is an increasing function of light intensity. The efficacy is also an increasing function of PS concentration (*C*_0_), as shown by Figure 13. When the oxygen inhibition effect is included, as shown by Equation (31), the above analytic formulas produced efficacy will be reduced. However, only numerical data are available. The modeling curves of Alvankarian and Majlis (2015) shown by their Figures 3F,G are not accurate because their Equation (5) for the conversion have over simplified the oxygen inhibition effect, shown by our Equation (17) by a reduction factor of (1 – [O_2_]) *C*_EFF_, and also assumed a constant PS concentration throughout the resin thickness. Therefore, their efficacy curves in their Figure 7G are overestimated, compared to our Figure 10, which includes the PS depletion.

**Figure 14:**
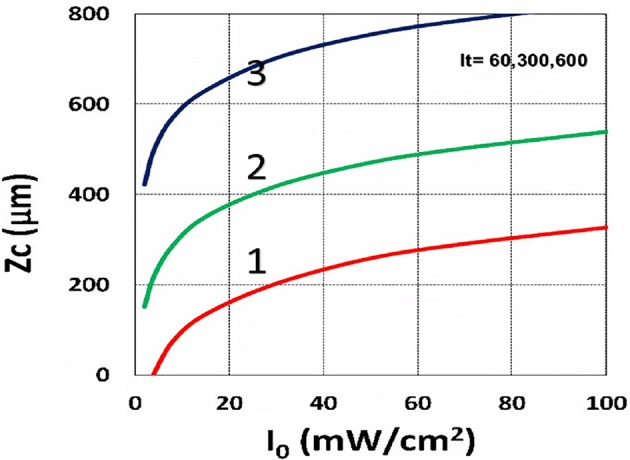
Curing depth profile. Curing depth (*Z*_*C*_) vs. light intensity (*I*_0_) for *t* = (60, 300, 600) s, for curves (1, 2, 3); for *C*_0_ = 0.01 mM, based on Equation (15) without oxygen inhibition.

### Curing Depth Profile

In microfabrication system, formation of radical (*R*′') decreases over depth (*z*) due to the reduction in light intensity and PS concentration and increase in oxygen inhibition. When oxygen diffuses into the film and consumes radicals, the balance of radical production and oxygen inhibition gives rise to the inhibition zone, where the polymerization is completely suppressed, as presented by kinetic Equation (37).

**Figure 15:**
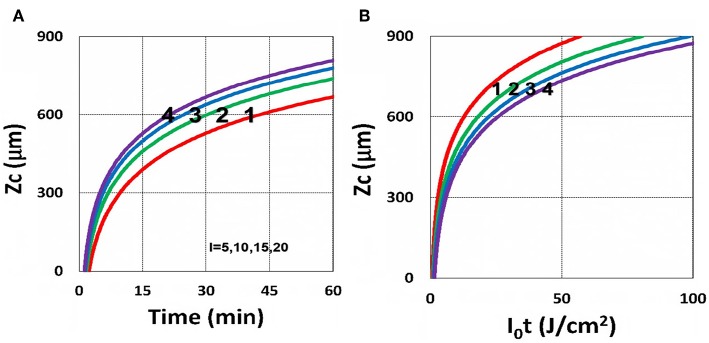
Curing depth profile. Curing depth for *I*_0_ = (5, 10, 15, 20) mW/cm^2^, for curves (1, 2, 3, 4); for **(A)** *Z*_*C*_ vs. time and **(B)** *Z*_*C*_ vs. light dose.

**Figure 16:**
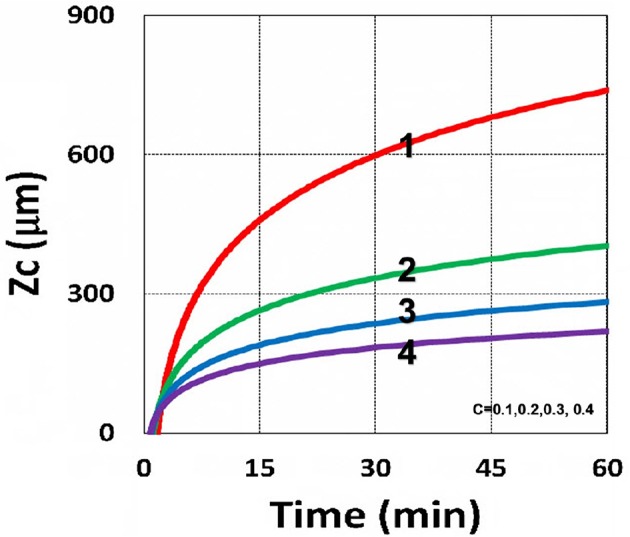
Curing depth profile. Curing depth vs. time, for *C*_0_ = (0.1, 0.2, 0.3, 0.4) mM, for curves (1, 2, 3, 4); for *I*_0_ = 10 mW/cm^2^.

**Figure 17:**
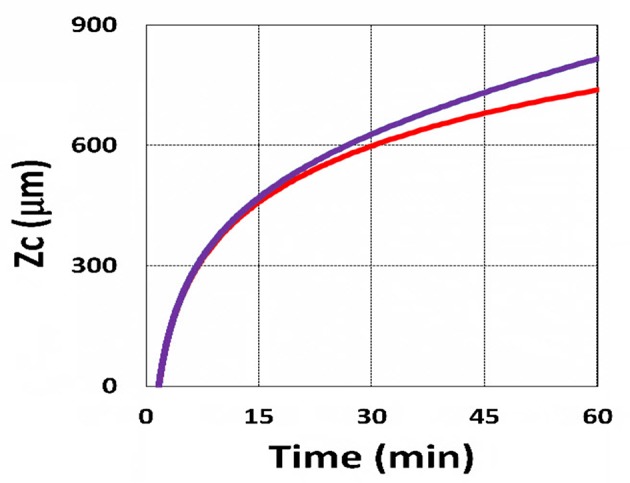
Curing depth profile. Curing depth vs. time, with (blue) and without (red) the correction of time-dependent absorption factor *A*′ shown in Equations (13) and (14) for *C*_0_ = 0.1 mM, *I*_0_ = 10 mW/cm^2^.

Using the analytic formula, Equation (37), we are able to investigate the roles of PS and oxygen concentration, light intensity, and exposure time in curing depth. Figure 13 shows that curing depth (*Z*_*C*_) is an increasing function of light intensity and exposure time. Figure 15 shows curing depth profiles vs. time and light dose, in which the intensity dependence is reversed in Figures 15A,B. This unexpected feature also shown in the efficacy profile, shown by Equation (35), that smaller light intensity achieves higher steady-state efficacy (for a fixed light dose). Figure 16 shows that curing depth is an increasing function of the PS concentration (*C*_0_), as also predicted by Equation (35) that efficacy has a scaling law of $C_{0}^{0.5,}$ and so does *Z*_*C*_. The effect of time-dependent absorption factor *A*′ shown in Equation (35) on the curing depth is shown in Figure 17 that assumption of a constant PS concentration, or *A*_1_*t* = 0 in Equation (34), will underestimate the cross-link depth 1–12%, especially for high dose and optically thick polymer. Figure 17 shows the viscosity effect on the efficacy. The effective rate constant (*K*) of Equation (35) is revised to a smaller value as: 1 – m [1 – exp(–*S*)], with *S* is the first-order solution (with no revision), where *m* = 0.1–0.3 is a fit parameter to measured data. The revised *K* leads to a smaller efficacy due to the less free volume of cross-link resulted by the increase of viscosity when efficacy increases.

It is important to note that the *S*-function is scaled by [*C*_0_*I*_0_*t*^2^]^0.5^ for transient state (with *B*′*t* << 1), and scaled by [*C*_0_/*I*_0_]^0.5^ for steady state. Therefore, the dose-dependence Bunsen Roscoe law, scaled by a light dose *E*_0_ = *I*_0_*t*, failed in a bimolecular termination system, with radical (*R*′) scaled by [*C*_0_*I*_0_ exp(–*B*′*t*)]^0.5^, as shown by Equation (35). These features also apply to the curing depth shown by Figure 13 that it is a non-linear function of time (*t*) and light intensity. The simple dose-dependence law does not apply to *Z*_*C*_. Figure 13 also shows that for a given light dose, higher light intensity achieves a lower depth, for large exposure time, with *B*′*t* >> 1. This leads to a strategy to use a higher light intensity for faster curing but an extended exposure time (~30%) than what is predicted by BBL is required to achieve the same curing depth as that of lower intensity. This strategy has been clinically demonstrated in corneal cross-link (Lin and Wang, 2016), but not yet in curing of other biomaterials.

The analytic formula of Equation (37) is available only when the oxygen effect is neglected, or *K*_12_[O_2_] = 0 in Equation (31). The oxygen inhibition effect on *Z*_*C*_ is further investigated by the numerical solutions of Equation (37), including *K*_12_[O_2_], where *Z*_*C*_ is graphically found by the crossing points of *C*_EFF_ = *C*_*T*_ (a threshold value, 0.8) and the curves of *C*_EFF_ vs. time for various *z*. **Figure 19** shows that *Z*_*C*_ is a decreasing function of external oxygen supply rate (*P*′) and the initial oxygen concentration, [O_0_], where larger [O_0_] or *P*′ lead to smaller efficacy, thus smaller *Z*_*C*_ due to larger oxygen inhibition effect, as also predicted by Equation (31).

### Analysis of Measured Data

Utilization of microfabrication to reduce the deposition steps and to obtain a monolithic product was reported by Alvankarian and Majlis (2015) and Chen et al. (2017), and Wu et al. (2018) in which structures of arrays of pillars in photo-cross-linkable films were measured by irradiation with a periodic array of microscale optical beams under ambient conditions. The optical beams experience a self-focusing non-linearity owing to the photopolymerization-induced changes in refractive index, thereby concentrating light and driving the concurrent, parallel growth of microscale pillars along their path length (Wu et al., 2018). In microfabrication system, formation of radical decreases over depth due to the reduction in light intensity and PS concentration and increase in oxygen inhibition. Under ambient conditions, oxygen diffuses into the film and consumes radicals. The balance of radical production and oxygen inhibition gives rise to the inhibition zone, where the polymerization is completely suppressed.

The curing depth (*Z*_*C*_) given by the solution of Equation (37) is proportional to the pillar height defined by Chen et al. (2017) where their measured data of their Figure 4 are analyzed as follows. Our Figure 13 shows *Z*_*C*_ vs. time based on Equation (37) that *Z*_*C*_ is proportional to ln (*t*^2^*I*_0_), for small *t*, and reaches its steady-state time (*T*_*S*_) (when *Z*_*C*_ = 0.9 h), which is scaled by $I_{0}^{-0.5}$. For example, our calculated *T*_*S*_ = (1,050, 735, 609) s, for *I*_0_ = (5, 10, 15) mW/cm^2^, which shows the similar trend of the measured data of Chen et al. (2017) *T*_*S*_ = (1,050, 600, 480) s. Furthermore, their measured pillar heights ranging 300–600 μm (at *t* = 600 s), for *I*_0_ = 5–15 mW/cm^2^ are consistent with our Figure 13. Figure 7 of Chen et al. (2017) also showed the similar trend as our scaling law of *Z*_*C*_ vs. light intensity given by Equation (35) that *Z*_*C*_ is scaled by (1/*A*′)ln(*I*_0_).

**Figure 18:**
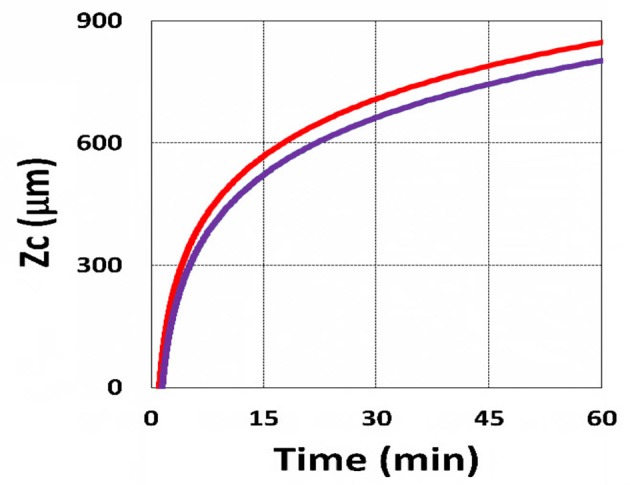
Curing depth profile. Curing depth vs. time, with (blue) and without (red) the correction of viscosity effect.

**Figure 19:**
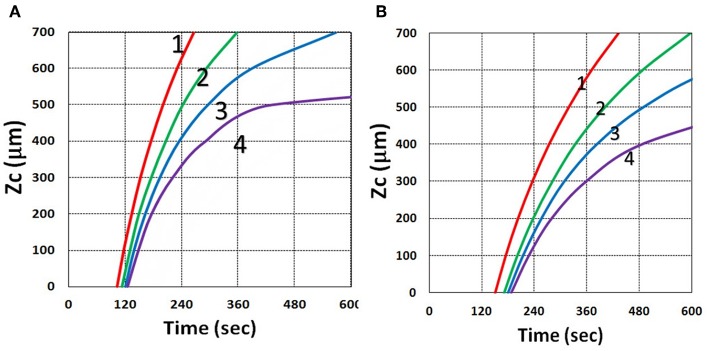
Curing depth profile. Curing depth vs. time, for various oxygen external supply rates, *P*′ = (0, 3.0, 5.0, 7.0) × 10^−6^ (mM/s), for curves (1, 2, 3, 4); for oxygen initial concentration **(A)** [O_0_] = 0.001 mM and **(B)** [O_0_] = 0.002 mM, for *b* = 0.0001, *C*_0_ = 0.02 mM and *I*_0_ = 10 mW/cm^2^.

Curing depth control is one of the key factors for microfabrication, in which the influencing factors of curing depth include light intensity and exposure time (or dose), the initial concentration of PS and oxygen, and the external supply of oxygen, as shown by Figures 13–19. For a given photo resin thickness of *H*, Chen et al. (2017) also defined an inhibition zone (*Z*_*N*_) given by *Z*_*N*_ = *H* – *Z*_*C*_. Therefore, a better understanding and manipulation of *Z*_*C*_ (and *Z*_*N*_) will enable a strong bias differential in the growth between bright (irradiated) and dark regions. The balance of radical production and oxygen inhibition gives rise to the inhibition zone. Figure 8 of Chen et al. (2017) showed a similar trend as our scaling law, in which the log–log plots of inhibition zone vs. light intensity show a linear relationship having slopes depended by various mask spacing ratios.

The above described experimental works have validated certain features of our modeling and the analytic formulas. However, further studies are required to validation other features presented by our modeling, such as (i) the role of PS concentration (*C*_0_) in thick polymers (>1.0 mm), which was assumed as a constant for optically thin polymers (<0.2 mm); (ii) the curing depth at various *C*_0_, as shown by our Figure 16; (iii) dynamic measurement of the light intensity in a strongly depleted PS system, which was assumed as time independent and follows the conventional BLL; however our, theory presented a non-BLL; (iv) as shown by Figure 18, the viscosity effect on the reduction in curing depth; and (v) the role of oxygen external supply rate on the reduction of curing depth, as shown by Figure 19.

### Strategy for Improved Efficacy and Depth

As discussed earlier, a higher light intensity will accelerate the curing speed but suffers a smaller curing depth and efficacy (at steady state). This also indicates that there is a limitation of maximum light intensity and the associate minimum exposure time. Higher PS initial concentration will improve the efficacy, but there is an optimal value (Lin et al., 2019). There are many conventional strategies to reduce oxygen inhibition in photoinduced polymerization: working in an inert or closed environment, increasing the photoinitiator concentration, increasing the light dose or light intensity (for reduced induction time), use of multiple photoinitiators with different rate of initiation, or addition of oxygen scavengers. Chemical mechanisms incorporate additives or suitably functionalized monomers which are insensitive to oxygen, such as the thiol-ene and thiol-acrylate-Michael additive systems (Claudino et al., 2016; Chen et al., 2019a,b). Additive enhancer monomer was proposed to improve the curing (cross-link) efficacy by either reducing the oxygen inhibition effect by stable monomer or increase the lifetime of radicals in clinical applications (Chen et al., 2019b; Wertheimer et al., 2019). The multimonomer system may be applied to industrial materials as an alternative to manipulate the microfabrication. Dual-wavelength (red and UV) photopolymerization was also reported, in which preirradiation of the red light eliminated the oxygen inhibition effect and thus enhanced the conversion efficacy of the UV light (Childress et al., 2019).

## Conclusion

Photopolymerization efficacy (*C*_EFF_) is an increasing function of PS concentration (*C*_0_) and the light dose at transient state, but it is a decreasing function of the light intensity, scaled by [*C*_0_/*I*_0_]^0.5^ at steady state. The curing depth is an increasing function of *C*_0_ and light dose (time × intensity), but it is a decreasing function of the oxygen concentration, viscosity effect, and oxygen external supply rate. For optically thick polymers, light intensity is an increasing function of time due to PS concentration depletion, which cannot be neglected. Saturation of efficacy profile is governed by the PS depletion rate and light dose. Efficacy is also an increasing function of the effective rate constant, *K* = *k*′/*k*_*T*_^0.5^, defined by the radical producing rate (*k*′) and the bimolecular termination rate. The curing depth is a decreasing function of the oxygen inhibition effect.

## Data Availability Statement

The datasets generated for this study are available on request to the corresponding author.

## Author Contributions

J-TL: concept. D-CC and K-TC: software and data. J-TL and H-WL: supervision. D-CC: financial. J-TL, H-WL, and D-CC: writing and editing. All authors listed have made a substantial, direct and intellectual contribution to the work, and approved it for publication.

### Conflict of Interest

J-TL was CEO of New Vision Inc. The remaining authors declare that the research was conducted in the absence of any commercial or financial relationships that could be construed as a potential conflict of interest.
